# A short history of academic general practice in UK medical schools 

**DOI:** 10.4102/phcfm.v5i1.463

**Published:** 2013-04-02

**Authors:** Derek A. Hellenberg

**Affiliations:** 1 Faculty of Health Sciences, University of Cape Town, South Africa

This book is intended for those interested in the historical development of general practice in the United Kingdom (UK). It gives a comprehensive account, from the establishment of the first department of general practice in Edinburgh to the development of other departments of general practice throughout the UK. It takes us on an interesting journey through the academic and political battles fought to attain the current status of academic general practice and also describes the many colleagues and characters who contributed significantly to this struggle. In addition, it has a chapter on each university that has a department of general practice and describes how their teaching, service and research roles have evolved over the years. The book would certainly appeal to those who enjoy history written in an easily readable style. 

This work represents the first attempt to collate the history of general practice throughout the UK into a single book. Each department of general practice faced various challenges when they started out and most of these would be familiar to all those involved in this discipline. A typical challenge, for instance, is resistance from colleagues in other specialities. It is instructive that most departments of general practice in the UK were located in Public Health or Community Medicine and had ‘champions’ in the parent discipline, as well as receiving support from the Royal College of General Practice and contributions by the National Health Service (NHS). The author gives examples of how some of the problems were overcome and these lessons would be particularly useful for those departments elsewhere who are still in the developmental stage of academic general practice.

The text presents evidence of how the teaching programmes in general practice evolved at the various institutions, where and how the teaching took place and how new and innovative teaching and assessment methods were introduced. It also tracks the development of research excellence in general practice and how this has had an impact in lifting the profile of the discipline. I found this information particularly helpful as we at UCT are still a relatively ‘young’ independent division of family medicine, being 10 years old!

The book is written in a style which is easy to read, with appropriate use of technical language where necessary. The layout of the text makes it readily accessible, with a clear contents page, list of abbreviations and a summary of the timelines in the development of significant events in the discipline. There are twenty-one chapters in all, and each of nineteen of them is dedicated to a different university. Chapter 6 describes Academic Practice in Ireland as a whole and Chapter 20 describes all the London Medical Schools. Each chapter includes the names of colleagues and others who played significant roles in lifting the profile of general practice in medicine. The book ends with four appendices which are relevant to the context of the book. The index provides a convenient reference to access the university which one is interested in as well as the names of specific people.

It is inevitable that some colleagues who contributed substantially to the development of the discipline in the UK were not mentioned in the book. Only brief references are made to the first chairs in Holland and Canada and perhaps there should have been a chapter on a global perspective on the development of general practice to allow one to make some comparison with the UK programmes. 

I would recommend this book to all teachers of family medicine, as there is a lot of useful information regarding the teaching methods used at the various medical schools, depending on the needs at the time. The book is informative as well as entertaining and I am definitely planning a second read! 

